# Soft Hands

**Published:** 1876-07

**Authors:** 


### Soft Hands.

To preserve the smoothness and softness of the hands, keep a small bottle of glycerine near the place where you habitually wash them, and whenever you have finished washing, and before wiping them, put one or two drops of the glycerine on the wet palm and rub the hands thoroughly with it as if it were soap, then dry lightly with a towel. Household work and bad weather will not prevent your skin from being smooth and soft if this plan of using glycerine is followed.

